# Community engagement for malaria elimination in contested areas of the Karen/Kayin State, Myanmar: A case study on the Malaria Elimination Task Force

**DOI:** 10.12688/wellcomeopenres.13965.1

**Published:** 2018-03-06

**Authors:** Andrea König, Ladda Kajeechiwa, May Myo Thwin, Suphak Nosten, Saw Win Tun, Decha Tangseefa, François Nosten

**Affiliations:** 1 Peace Information Center, Faculty of Political Science, Thammasat University, Bangkok, Thailand; 2 Shoklo Malaria Research Unit, Mae Sot, Thailand; 3 Faculty of Political Science, Thammasat University, Bangkok, Thailand; 4 Centre for Tropical Medicine and Global Health, Nuffield Department of Clinical Medicine, University of Oxford, Oxford, UK

**Keywords:** Community Engagement, Malaria Elimination, Myanmar, Border, War

## Abstract

**Background:** This study evinces how the community engagement (CE) approach of the Malaria Elimination Task Force (METF) in the Karen/Kayin State of southeast Myanmar contributed to generating participation in program activities, such as early detection and treatment, malaria blood surveys and mass drug administration.

**Methods:** We investigated the CE approach through ethnographic and key-informant unstructured interviews as well as focus group discussions and participant observation with program participants and METF team members. Interview transcripts were analysed manually applying inductive content analysis.

**Results:** In the context of the Thai-Myanmar border, CE had two main dimensions: The first was to enable the politically sensitive entry of the program into intervention areas, the second was to create mutual understanding among the METF team and people from the population and gain trust. For the first, the METF needed to acknowledge, get permission and communicate with a complex landscape of governing actors and leaders. For the second, creating mutual understanding meant to spend time learning about villagers’ lifeworlds and adapting engagement accordingly to create understanding of the program in resonance with these lifeworlds. Gaining trust meant to leverage leadership and build trust through informal “horizontal” engagement and acts of caring. Three key dimensions of villagers’ lifeworlds that appeared most crucial for staff to learn about were: villagers’ experience with the outside world; leadership and governing relationships to villagers: authority and distrust; and their perceived need for malaria care.

**Conclusions:** Successful CE is highly context specific. CE success was based on the adaptability of the approach towards the complex political and socio-cultural contexts of the different areas of the Karen/Kayin State. Hence, CE is not a fixed approach or strategy defined before entering the setting, but constantly evolving based on the encounters of ff with stakeholders and people of the population.

## 1 Introduction

### 1.1 Malaria elimination along the Thai-Myanmar border

**Figure 1.**
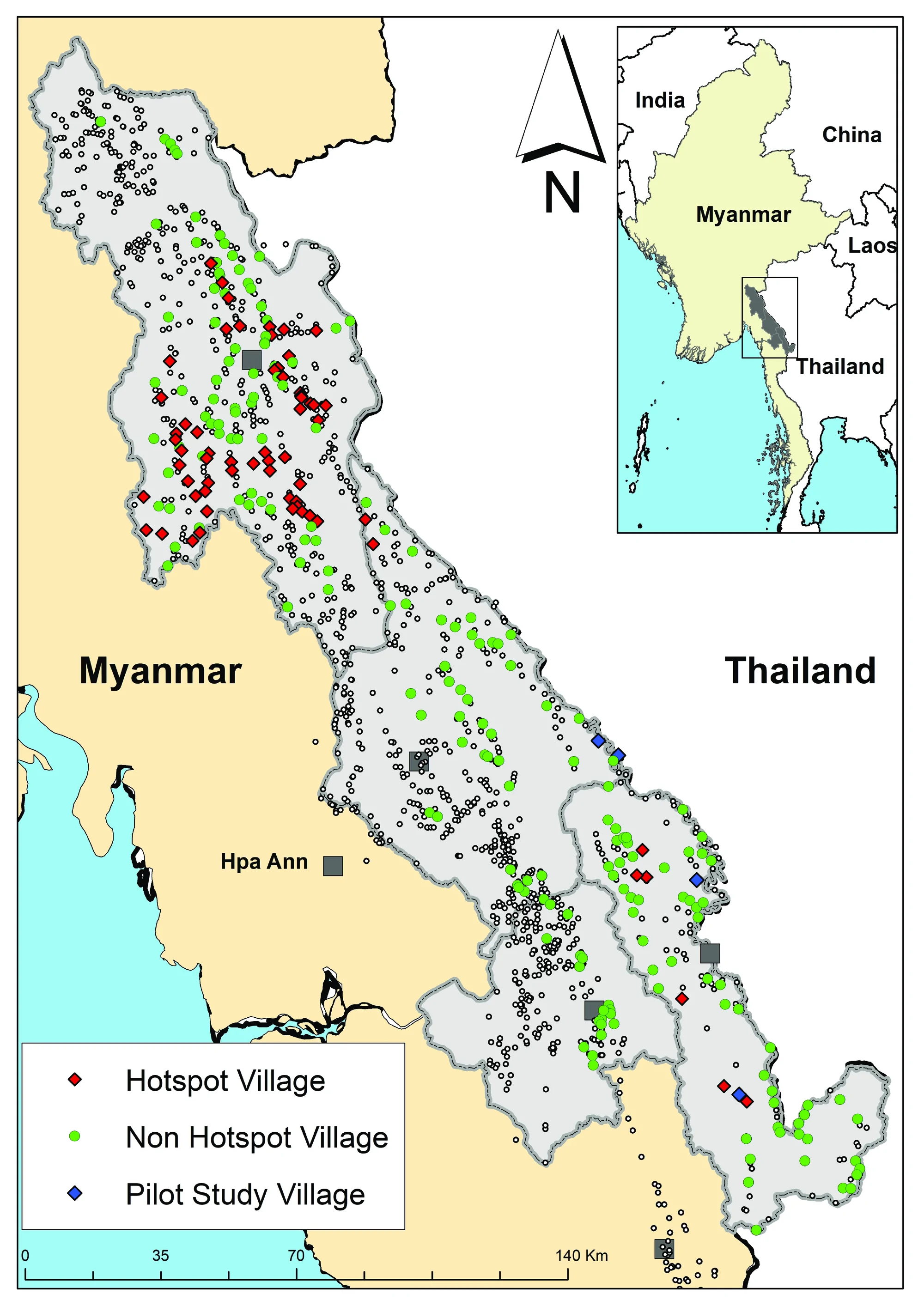
METF program areas one, two and three from north to south with mapped villages. Source: this map was provided by the METF with the permission to display it in this publication. It was produced by MyoChitMin.

The Karen/Kayin state lies along the Thai border. People living along the Thai-Myanmar border show great ethnic, linguistic, religious and political heterogeneity and a high degree of mobility due to displacement, labour migration and the so-called refugee camps along the border ( Smith, 2017; Tangseefa, 2006). The people of the program’s population however mainly belonged to the Karen/Kayin ethnic groups. The total population living in 1222 villages, mapped by the Malaria Elimination Task Force (METF) within the three program areas in the Karen/Kayin State (see Figure 1), was estimated to amount to 365,000 ( Landier *et al.*, in press). The METF program area was divided into three areas from north to south, which were again divided into zones. For each area there was an area coordinator and for each zone a zone coordinator ( METF, 2016). For more details on the METF organizational structure see Figure S1 and Figure S2.

As the Thai-Myanmar border is an area with a history of war and intermittent fighting, access to health care is still grossly deficient ( Low *et al.*, 2014). As Davis & Jolliffe (2016) summarise:

*Since Myanmar’s independence in 1948, the southeast region has been torn by armed conflicts between the government and multiple ethnic armed organizations (EAOs) calling for greater autonomy in their regions and a more equal stake in national affairs. These conflicts have had deeply harmful impacts on health in the region, and have led to a deeply fractured governance environment that greatly impacts the ways that healthcare is delivered, and by whom, depending on territory. (p. 4)*

Myanmar has a high malaria burden and this burden is exacerbated in areas where access to healthcare is deficient ( Smith, 2017). Moreover, emerging artemisinin-resistance of *Plasmodium falciparum* malaria is posing a major threat to malaria control. The resistance is increasingly prevalent in Southeast Asia, including the Thai-Myanmar border. As resistance could spread through Asia to Africa, efforts have been made to eliminate *P. falciparum* ( WHO, 2015). In order to stop resistance spread from the Thai-Myanmar border, the METF was founded in 2014 to eliminate *P. falciparum* malaria in this area. The METF is a consortium of local health care providers, such as ethnic and community-based health organizations, who have provided health care services to border populations since the late 1980s ( METF, 2016). The METF received technical support from the Shoklo Malaria Research Unit (SMRU). The SMRU has been involved in providing healthcare and conducting operational research in the border population for thirty years ( http://www.shoklo-unit.com/). To progress towards elimination, the METF employed seven program components, including (1) Mapping and a geographic information system, (2) Community engagement, (3) Malaria posts (MP), (4) Real-time data collection and reporting, (5) Submicroscopic malaria prevalence surveys, (6) Mass drug administration (MDA) and (7) Entomology ( Parker *et al.*, 2017). As of the end of 2017, the METF had opened MPs in 1222 villages, conducted surveys in 272 villages, identified 69 hotspots and conducted MDA in 61 villages. The METF had achieved a median participation of 89% among villagers. The impact of this program on malaria has been profound: the incidence of clinical cases of *P.falciparum* has declined by 60–98% and 965 out of 1222 villages have reached the elimination threshold as defined by the World Health Organization (WHO). This is attributable mainly to the network of functioning MPs, MDA playing the role of accelerator in the elimination ( Landier *et al.*, In Press).

### 1.2 Community engagement in the Malaria Elimination Context

Community engagement (CE) has been recognised by the WHO as crucial to the success of malaria elimination and control interventions, in particular in the fight against artemisinin resistance ( WHO, 2015). CE is key to malaria elimination, as a very high percentage of the population needs to participate in MDA for it to be successful. Even a small number of non-participants can undermine the elimination success ( Cheah & White, 2016).

There are various definitions of CE circulating in international health research (see examples in Tindana *et al.*, 2007). Hence, any health program needs to clarify what its CE approach entails. A broad definition is given by the Centers for Disease Control and Prevention (CDC). According to the CDC, CE is:

*A process of working collaboratively with and through groups of people affiliated by geographic proximity, special interest, or similar situations to address issues affecting the wellbeing of those people ( CDC, 1997, p. 9).*

The METF CE approach lies within this definition, as the target population was located in one geographic area, the Karen/Kayin State in southeastern Myanmar, and affected by the threat of emerging artemisinin resistance and malaria in general. While CE literature often refers to a long-term partnership with communities for sustained and general health improvement, malaria elimination is primarily motivated by the *global* public health priority to stop drug-resistance spread ( Atkinson *et al.*, 2011). Hence, two aspects of CE, namely assessment of needs and identification of solutions by the affected community, were already determined by the global threat of drug resistance and its urgency when approaching the population. The purpose of CE under these preconditions was to facilitate the ethical and medically successful implementation of the METF’s elimination activities in accordance with the communities, local Karen ethnic armed organizations (hereafter KEAOs) and the Myanmar Ministry of Health.

While the importance of CE for malaria elimination approaches is widely acknowledged, literature on the role and nature of CE in such contexts is scarce ( Adhikari *et al.*, 2016; Sahan *et al.*, 2017). However, quite a number of studies on CE for malaria elimination in Southeast Asia and also Myanmar have been published in 2016 and 2017. Kajeechiwa *et al.* (2016) report in their quantitative study on four piloting villages of a targeted malaria elimination (TME) program, which would later become the METF, that participation was challenged by a lack of understanding of the intervention. Moreover, they observed that participation in MDA was higher in two cohesive communities with similar ethnic and demographic backgrounds and lower in a particularly conflict-torn and a fragmented village. In another publication on their CE efforts, challenges faced and lessons learnt, Kajeechiwa *et al.* (2017) emphasise the importance of being flexible related to a lack of infrastructure and communication networks, the importance of adaptation towards villagers’ seasonal activities, engagement of children through schools, adapting explanations towards villagers’ lack of education, be aware of local politics, be prepared to answer health questions and deal with rumours and misunderstandings. In their qualitative study on CE in the same pilot villages, Tangseefa *et al.* ^1^ (unpublished report) developed eight dimensions of people’s lifeworlds that were important for CE in the context of the elimination program: (1) The space people lived in, (2) the history of the village, (3) what kinds of work people did, (4) whether they subscribed to a capital logic, (5) their knowledge about the world, (6) what leadership figures were associated with the village, (7) villagers’ relations with health institution(s) and (8) whether there were any rumours going around in the village. In their study on CE for targeted malaria treatment (TMT) in other parts of the Karen/Kayin State, Sahan *et al.* (2017) write that *“Community engagement was seen by staff as integral to the TMT project as a whole and not a sub-set of Activities*”. Furthermore, they emphasise the importance of building trusting relationships and the benefit of offering healthcare alongside TMT. Time constraints, isolation of communities and the history of conflict were mentioned as challenges for CE.

As clinical encounters are always some forms of social exchanges, we decided to not limit our focus to designated CE activities and considered CE as part of each interaction between the program and the people it engaged with ( Carruth, 2016). In our first study ( Tangseefa *et al.*, unpublished report), we learnt that CE workers had to be aware of ways in which varieties of peoples understood their own quotidian lives in relations to others. In this light, Jürgen Habermas’ concept of lifeworlds – which is the sphere of understandings that are informal and culturally-grounded ( Habermas, 1984) – was a helpful point of departure for our first and present study. However, because Habermas emphasises mutual accommodations among actors in a public space, we had to deviate by opening up to contestation due to: the lingering contesting sovereignties and peoples’ memories of hatred and violence. After Tangseefa *et al.* had conceptualised eight dimensions of people’s lifeworlds that mattered to CE in their study, we now focused on how the ways of engaging *responded to* those lifeworlds ( Box 1). Based on the CE activities described by METF team members and their reasoning why they conducted these activities, we tried to understand how they influenced villagers’ decision to participate related to their lifeworlds. We chose to look at this relation between CE and lifeworlds in order to understand what constitutes a successful CE approach in the context of the Thai-Myanmar border. We only focused on the short-term dimensions of CE success, as long-term benefits of the CE efforts will become visible only later on -- being aware that sustainable health improvements of communities are an important goal of CE.

Box 1. **Eight dimensions of people’s lifeworlds ( Tangseefa *et al.*, unpublished report)**

(1) the **space** people lived in
(2) **history** of the village
(3) **kinds of work** people did
(4) whether villagers subscribed to **capital logic**
(5) villagers’ **knowledge about the world**
(6) **leadership** figures associated with the village
(7) villagers’ **relations with health institution(s)**
(8) **rumours** going around in the village

In summary, our study tries to evince: How did CE for malaria elimination generate high program participation in resonance to people’s lifeworlds and in the context of the program area, which was characterised by inter- and intra-ethnic heterogeneity, a political transition and a complex and insufficient healthcare landscape?

## 2 Methods

The study was conducted as a case study as it was commissioned by the METF with the motivation to investigate what had made its CE approach successful. It followed an ethnographic approach in order to understand how CE related to the lifeworlds of people in the program area. The research team included a social scientist, a global health specialist and a medical researcher, with the aim to bridge between the medical program context and the ethnographic research question. CE specialists from the METF team facilitated and organised the research activities. As the study was a continuation of a former study, some of the researchers were already experienced with the research context and focus. Data collection was conducted from April to August 2016 and data were analysed in parallel with data collection until the end of August 2016. Ethnographic and key-informant unstructured interviews (N = 38) as well as focus group discussions (N = 2 with 4 and 5 participants respectively) were conducted with program staff from the three METF areas about their work *.* Moreover, program participants (villagers) were interviewed individually (N = 7), as well as in focus groups (N = 3 with 2, 4 and 3 participants respectively) during two field trips to villages. Interviews were conducted in English with a Karen/Kayin and Myanmar speaking translator and in one case in Thai. None of the interview participants were provided any allowances for participation.

Interviews with METF team members were unstructured beginning with the request to describe their CE work for the METF, with follow-up questions whenever they talked about a topic relevant to CE. A question usually asked during the interview was what challenges they faced in generating participation and how they addressed these. The foci of interviews were adapted based on evolving themes from the analysis of former interviews. The purpose of adaptation was to create a comprehensive understanding of different dimensions of CE. Guiding interview questions ( Supplementary File 1) for short interviews (5–30 min) and FGDs with participants of the METF program addressed a) their reasons for participation, b) any initial concerns about participation and c) their experiences with the program. Participant observation was conducted during METF meetings and three field trips (with a duration of 2 days, 1 day and 2 days respectively) to three villages in the program area to observe program activities. Purposive sampling was applied to include various perspectives within the METF program. Convenience sampling was applied for team members and particularly for villagers, as not all program areas were equally accessible for foreigners due to restrictions by local authorities or security concerns. Sampling was ended after a time limit had been reached. Oral informed consent was obtained for each interview that did not emerge from an informal conversation. Oral consent was chosen over written consent due to the procedure of written consent being potentially intimidating for interviewees. Many of them had been holding a health or political position in former war zones and some had been subject to interrogation in the past. This also concerned villagers interviewed for the study. In order not to resemble the atmosphere of an interrogation, we tried to create a more informal, comfortable atmosphere. Interviews with team members were audio recorded with their consent. Interviews and FGDs with program participants were not recorded in order to maintain a conversation-like atmosphere. Due to time constraints, records were transcribed into condensed notes. The interview transcripts were anonymised and numbered, with participant information together with the numbers being saved on a password-protected computer in a separate file.

Interview transcripts and field notes were analysed simultaneously with ongoing data collection in cooperation of two researchers, AK and DT. Themes were developed through inductive content analysis, manually, based on the analysis of each field note and interview transcript. To enhance credibility, preliminary themes were presented to the METF (CE) team for checking and feedback and consequently revised.

Any interview excerpts, examples and interpretations in our results that are relating to CE in the context of MDA are drawing on experiences from only 51 MDA villages among 1040 METF villages in which a MP had been opened at that time of data collection.

### 2.1 Ethics

This work was part of a larger project on malaria elimination approved by the Ethics Review Committee on Medical Research Involving Human Subjects from the Republic of the Union of Myanmar, Ministry of Health and Sports, Department of Medical Research (Lower Myanmar): 73/Ethics 2014.

## 3 Results

### 3.1 Community engagement with governing actors and leaders: Navigating transitioning power relations

The METF operated in a politically and socially transitioning setting with intermittent fighting in some areas. CE in this context was inherently politically sensitive. On the governance and leadership level, the METF needed to engage the Myanmar government, various KEAOs and informal leaders in order to obtain permission and authority to conduct the program in their territories. Sensitivity to sources of conflict was crucial in navigating contesting actors.

The METF program area included mostly remote or politically restricted territories, where control and legitimacy by the central government remained limited. Contestations consisted among Karen/Kayin armed groups and between different factions within armed groups, who adopted different policy positions. While staff reported that these groups had increasingly opened their territories to outsiders since the nationwide ceasefire agreement (NCA) in 2015, it was still crucial to obtain permission and inform them before conducting any activity in their territories. Doing so was complicated, as some authorities would even reject a meeting with the METF. Contact could often only be established through middle or mediator persons, for instance religious leaders. Hence, when meeting local leaders, it was critical that the staff maintained political impartiality and focused on the mutual health goal. As one area coordinator stated:

*“During the meeting with armed group leaders I try to control my emotions. Sometimes we disagree in political or religious issues, but each party tries to be careful not to trigger each other’s anger and keep their own point of view to themselves. It’s not the priority. We talk only about community needs...and avoid political topics.” (Area coordinator, m, 29).*

Moreover, governing functions among local authorities of KEAOs were highly decentralised and the authorities were often not well-connected throughout the administration line or disagreeing among different bodies of a KEAO. In such cases, the METF had to approach each level or faction separately to obtain permission to enter a new area locally. Where authorities were well-connected, those on top could facilitate the contact further down.

After the METF had approached authorities individually, it held inclusive stakeholder meetings with all local authorities and obtained mutual agreements on conducting the program in their territories to prevent confusion and conflict among all actors. Such meetings could be the first time that actors of the different KEAOs in a territory came to one table to address a shared health problem. Such meetings were important, as another source of small scale conflicts in those areas was misunderstanding. The meetings were not only for the METF to have all the actors consenting to the same “rules” for the program to enter, but also for the actors themselves to see their fellow and competing leaders agreeing to the METF. Seeing their agreement ensured them that they would not breach with other authorities by giving their permission. METF staff facilitating this entry CE phase of the program had to be locally respected persons, often they were leaders of KEAOs’ health departments or in charge of clinics run by armed organisations. Some of them enjoyed good relationships with different armed organizations due to their focus on health. One particularly well-connected zone coordinator exemplified this by stating:

*“The meeting [with local authorities] is just for informative purpose, they know me all and will agree anyway, I do not have to ask for permission, but just show respect through informing and acknowledging them.” (Zone coordinator, m, 51).*

The statement shows that *acknowledging* leaders and *asking for permission* were important to acknowledge them as the legitimate leaders of the territory that the METF wanted to enter. Firstly, the METF obtained an official permission from the Karen Department of Health and Welfare (KDHW) to enter and implement the program in Karen National Union (KNU) territory. The collaboration with well-connected actors was inherent in the makeup of the METF as a *task force* constituted of locally established health service organizations and health authorities with existing trust and working relationships with armed groups. The METF zones therefore were determined by the spheres of influence of the respective zone coordinators and ended where their influence ended.

The Myanmar government represented another key political stakeholder. Firstly, the METF was dependent on the government’s permission to conduct the program and receive funding from international donors. Therefore, advocacy with the government was an important aspect of CE.

For EAOs it was a critical time to negotiate their governing roles within the Karen/Kayin State for the future. The range of strategies pursued among Karen health leaders towards the central government sometimes created uncertainty about how to position themselves towards the METF program, which could cause tension:

Case 1 - One medic of an armed group controlling one program area was ambitious to build a close relationship with the government and attempted to establish malaria care in his area through an INGO working through the government. Hence, he felt his interests undermined by his co-medic, who supported the METF as a zone coordinator. He consequently started accusing the METF associated medic of being a traitor to the armed group by opening the area towards other groups, which temporarily caused the zone coordinator to lose trust among some in his armed group. (Zone coordinator, m, 41).

That the METF was working under a memorandum of understanding (MoU) with the government was hence important for the Karen/Kayin METF member organizations. As local Karen/Kayin authorities were careful not to breach with their parallel Myanmar counterparts, most METF staff informed local authorities of the MoU when introducing the program and invited them to represent the program together with Karen/Kayin authorities when introducing it to village leaders.

In summary, the METF embraced different policy positions through: the MoU with the Myanmar government; official permission from Karen ethnic leaders; and program ownership by KEAOs’ health departments as well as community based health organizations.

### 3.2 Community engagement on the village level

After obtaining permission on the governance and leadership level, the METF staff engaged on the village level. The main aspects of CE on this level were to create mutual understanding - for the team about the villagers’ lifeworlds and for villagers about the program - and generate trust. Two quotes from interviews with staff summarise these core points:

*“No CE is like riding a horse without knowing if it is a mare or a stallion, or like a duck in the water not knowing if it is deep or shallow - your effort will be useless.” (Zone coordinator, m, 35-40).*

*“It [the reason for rejection of the METF program] is either misunderstanding or distrust.” (Survey team member, m, 37).*

The two statements show two barriers to program success, which CE was addressing: a) lack of mutual understanding and b) lack of trust. To create mutual understanding, the team needed to learn about the communities in order to know how to approach villagers and adapt their way to create understanding of the program. To gain trust, they needed to leverage the trust of leadership and governance bodies or build trust through horizontal informal engagement. Three key dimensions of villagers’ lifeworlds appeared to be most crucial for staff to learn about. Those were: villagers’ experience with the outside world; leadership and governing relationships to villagers: authority and (dis-) trust; and villagers’ perceived need for malaria care. In the following, we illustrate these characteristics and how the CE approach responded to them.

***3.2.1 Experience with the outside world.*** Due to the geographical or armed group enforced isolation of some areas or villages, team members often reported that villagers had been isolated from changes in the outside world, such as a political transition or the practice of biomedical health care. Such isolation could cause fears among villagers regarding political or health consequences of participation, which were reasonable based on their past experiences of violence and confusion caused by some health programs. Hence, it was important that METF team members learned about villagers’ lifeworlds and created understanding of the program activities, basic concepts of malaria, as well as seasonal diseases. To be able to respond to fears related to participation immediately, it was important to be present, explain, care, cure and resolve rumours. Distrust was amplifying the importance of creating understanding and preventing misunderstanding.

*Isolation*

Villagers in some areas were isolated from the outside world due to the lack of (all-season) roads, limited communication networks and hardly any electricity. Moreover, some of the territories were not accessible for outsiders due to restrictions by local authorities. Travel to these places could be difficult and time-consuming. Moreover, some paths were heavily mined, which posed a security risk. The less exposed people had been to changes taking place in the outside world (such as the country’s socio-political transitions, development projects and biomedical health care) the less comparative experiences they had to make sense of the METF. That is, the less did the program make sense within their lifeworlds. As one CE worker stated:

*“The more they are isolated, the more vulnerable they are, the more they stick to their traditional beliefs.” (CE worker, f, 55).*

In some villages, people had never taken pills or had their blood taken before. Hence, many were afraid of these procedures. Some feared that the blood loss would make them weak or that the pain would be unbearable. The staff tried to take their fears to make them feel safe, by making connections to experiences from people’s daily lives (see section 3.2.1, *Ways of communication*).

Isolation was not only related to spatial remoteness and lack of infrastructure, but also described by some staff as an attitude of villagers, who avoided any contact with outsiders and only wanted to be left alone. The description of team members resembled Scott’s characterization of hill peoples, who avoided oppression and exploitation by governing actors through their hidden way of life ( Scott, 2009). In this sense, isolation was closely related to distrust. In such villages, people often relied on trusted leaders and persons from the village, who had been to the outside, for their judgement about the program (see section 3.2.2).

Moreover, some team members mentioned education to determine people’s understanding of the program. However, as education was often associated with leaving the village and studying in towns or the so-called refugee camps on the Thai side, it is possible that experience in such places of the outside world was more important for understanding than the education available in the program area. As one CE worker explained:

*“If they just stay in the village, whether they have no education or study until high school, that doesn’t make much difference. But if they have been to the outside, then they understand much easier what we are talking about.” (CE worker, m, 48).*

Villagers’ experiences with the outside world also related to their health beliefs and what cultures of healing they subscribed to.

*Health beliefs and cultures of healing*

Traditional health beliefs were, very common among villagers, not only in remote villages, but also in more connected lowland areas. In places more exposed to the outside world, people followed hybrid forms of traditional and modern healing practices. Program staff stated that when they were confronted with health beliefs differing from the biomedical conceptualization of the METF, it was best to acknowledge those beliefs and try to communicate the program in a way that harmonised with local beliefs. One CE coordinator stated:

*“..., if a person experiences or imagines side effects, we “heal” those side effects in their traditional way, we have to find someone in the village who can do that. In one village a local healer did magic to a glass of water and gave it to the person to drink, after that, the person felt better.” (CE worker, m, 57).*

To be able to adapt their way of engaging with villagers, it was helpful for the CE team to be familiar with various health beliefs in the area. People conceptualised, interpreted and treated seasonal diseases according to their health beliefs. Before conducting MDA with its potential side effects, the team talked to villagers about present seasonal diseases and their symptoms, in order to prevent them from conflating disease related conditions with side effects of the MDA drug. If that happened, rumours could spread that the MDA drug was causing the symptoms. Rumours that emerged about the program could be detrimental to participation. Besides holding information sessions on seasonal diseases, the CE team decreased the risk of rumours through to their “OPD” (Out Patients Department) component. OPD staff went and treated sick people in the village before MDA, so that disease symptoms were not confused with side effects of the MDA drugs. The OPD care also allowed the METF team to respond to side effects immediately. Moreover, it was important to simply be present wherever a confusion emerged, so that the team could immediately respond to fears about disease symptoms and MDA side effects, before a rumour spread. Therefore, it was crucial to stay 7 days from the first MDA day, the period during which side effects could occur. Regarding rumours, it was helpful if the CE team was experienced with recurrent rumours, had confidence and good argumentative skills. Some staff showed this confidence by talking with villagers about common rumours before the program in order to prevent them being started by someone in the village. In light of the suspicion that many villagers held towards the program, the CE workers needed to appear credible and proficient in what they were doing.

*Credibility*

Many staff emphasised that a very deep understanding of the medical and programmatic aspects of the program was a strong foundation for doing CE work. If villagers could not estimate the risks of participating, they at least needed to get the feeling that the METF team knew what they were doing. For concerns and questions about participation in program activities, the CE workers functioned as mediators between the medical staff and the villagers. In order to constantly improve the CE team’s ability to answer any questions, the METF held follow-up workshops after each round of MDA, where difficult questions were collected and answered. The METF made immense efforts to provide biomedical education to health workers and students to enable them to develop an in-depth understanding of the malaria parasite, pathology, transmission and treatment. Providing local health workers with such a knowledge base empowered them to be the agents in the fight against drug resistance.

In some places the CE team’s credibility could also be enhanced by making associations with *international, professional* or *modern* institutions and medical treatments. CE workers reported that to do so, they mentioned the METF academic partners, such as the associated universities, as well as all the METF member organizations. Some staff also told villagers that the MDA drug was recognised by the WHO as an international organization. Besides efforts to create a credible image, the role of CE was to create mutual understanding and communicate in a way that made sense within people’s lifeworlds. Consequently, team members needed to either already be familiar with, or learn about people’s lifeworlds.

*Learning about villagers’ lifeworlds*

To be able to adapt their engagement and program activities to villagers’ lifeworlds, the CE team needed to gather information about the village they would approach in advance. Some did not actively need to do this, as they were chosen based on the relationship they already had to the village. If they had a more distant relationship or none at all, they tried to meet local leaders in advance to get information about the village. If this was not possible or insufficient, CE members conducted “horizontal” engagement activities (also see section 3.2.2) that helped them get to know the villagers. Through such activities, the CE team tried to learn about the eight dimensions (see section 1.2) in the village. ( Tangseefa *et al.*, unpublished report).

Based on the understanding the CE team had developed of a village, they adapted their engagement and the implementation of program activities. To create understanding of the program, they used the following ways of communication in mass meetings, small-group meetings and house visits: simple language, visual materials, telling stories and comparisons. The techniques chosen depended on what stories or comparisons were familiar to villagers.

Ways of communication

a) Simple language

When talking about the program, many CE team members emphasised that they used simple language or wording:

*“When I talk about the program to the villagers, I do not use technical words, for example I do not say falciparum malaria, I call it ‘the dangerous malaria which kills people most’, I also avoid words like ‘MDA’, ‘CE’, ‘hotspot’, ‘qPCR’ or ‘survey’.” (Area coordinator, m, 29).*

b) Visual materials

**Figure 2.**
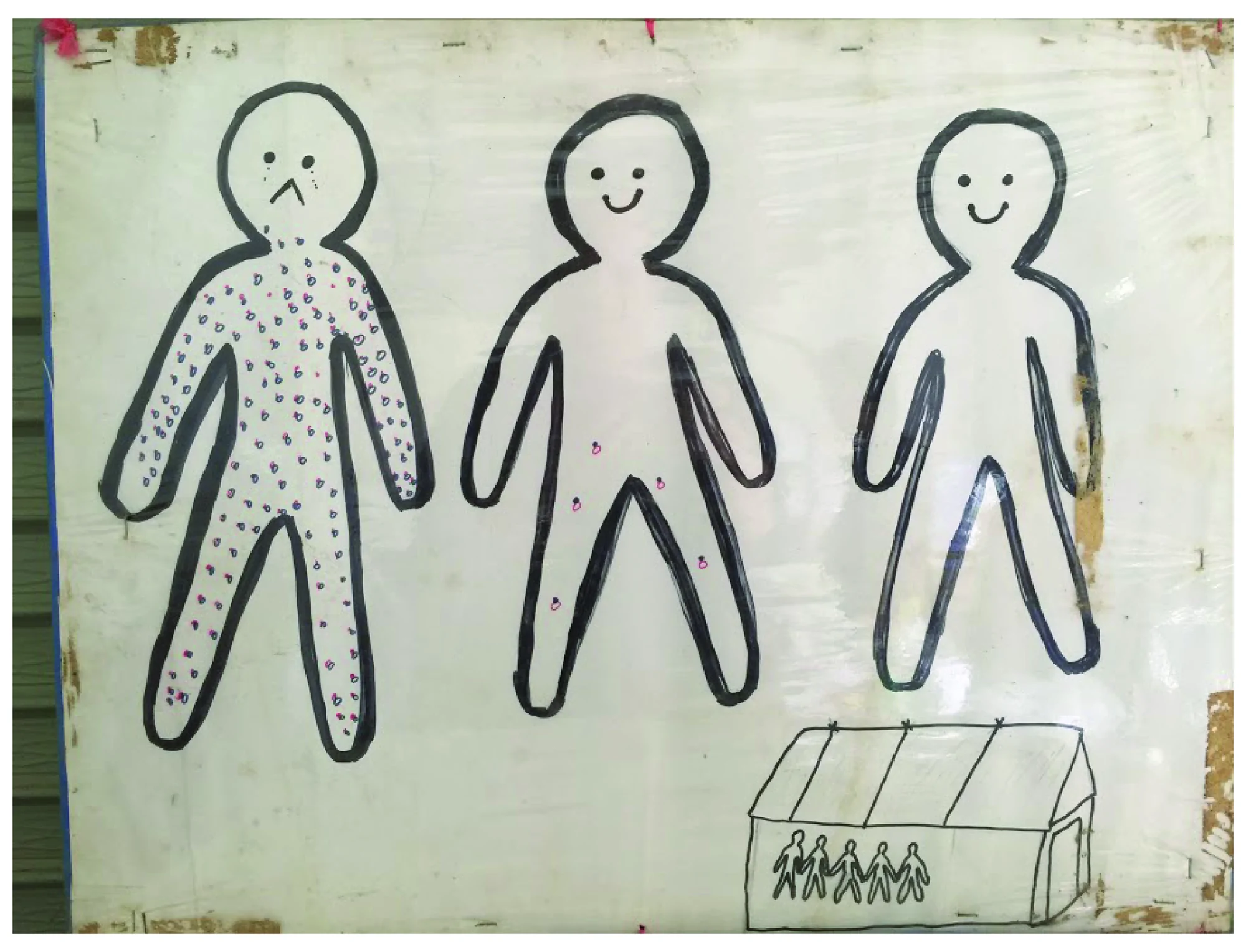
A handmade teaching material about asymptomatic infection with malaria. Source: METF CE team, provided with permission to display in this publication.

To provide basic understanding of the medical concept of malaria, such as the life cycle, transmission, and the MDA drugs, program staff used simple, visual materials such as cartoons and handmade drawings. To understand these materials, people did not have to be literate. Complex concepts like asymptomatic infection could be made perceptible through such visualizations (see Figure 2). Moreover, CE workers reported that villagers sometimes found the simple drawings funny, as they were not looking very formal and exposing the limited artistic skills of the CE workers.

c) Telling stories

Stories were a powerful medium to embed the program in villagers’ lifeworlds. A story could build a connection between villagers’ past and present, through which the program would make sense in the present without conflicting with past values:

Case 2 - One CE coordinator connected artemisinin back to the past of the Karen people. As he knew that the plant from which artemisinin was extracted had been used in China since long time ago, he indirectly linked the medicine to the identity and heritage of the Karen people, by explaining that it had been used since the time when ‘Pu Taw Mae Pa’ lead the Karen people down from Mongolia to the nowadays Myanmar. Most people knew this tale of their movement to nowadays Myanmar. (CE coordinator, m, 48).

Sweet wormwood ( *Artemisia annua*) has indeed been used in China in the past to treat some fevers before Artemisinin was discovered in a research program starting in 1967 ( Faurant, 2011). This story connected the unknown MDA drug to something the villagers knew and identified with, and also implied that the drug had been used since a long time and approved by places beyond Myanmar. Which stories the staff told, depended on the stories and tales that were well known in a village.

d) Comparisons

Another way to demystify the program was to compare the unknown program contents to an example borrowed from villagers’ lifeworlds, something that was familiar to them. To make participants understand that taking blood would not lead to an irreversible reduction of the amount of blood in their bodies, one survey team member told them:

*”Taking blood from your body is like picking a vegetable from a plant. You pick the vegetables, but they always grow new. When taking blood, the same happens, blood is always rebuilt after blood loss.” (Survey team member, m, age unknown).*

Through choosing attractive and understandable ways of communication, the program was demystified and became more tangible. Answers were provided to questions and concerns people had. On a general level, these parts of CE also enabled participants to become more familiar with the team and the program.

In summary, creating understanding about the program did not mean to provide a full education on biomedical concept of malaria, but information that was relevant to the concerns of people related to participation. Providing information and making the effort to create understanding had the secondary effect of showing that the program made an effort for the people. Being given a chance to understand and agree to the program was a positive experience for villagers, as this was not common among local leaders. Usually people were expected to follow leaders’ requests without much explanation. Hence, it may have caused villagers to feel thankful towards METF team members and participate in return.

***3.2.2 Leadership and governing relationships to villagers: authority and (dis-) trust.*** To approach a village for introducing the program, the METF teams were leveraging leadership ties to villagers, wherever those existed. Where few or no leading figures were in place or where they lacked authority and/or trust, the CE approach had to come horizontally, through informal personal engagement. Also helpful for generating trust were activities that entailed caring. Moreover, enough time and repetitive approaching were crucial to building and sustaining trust.

*Distrust and lack of affiliation with leaders and governing actors*

In the context of a history of war and intermittent fighting, fear of governing actors such as armed groups was a common theme. People still felt threatened, when armed groups appeared, as these groups had been associated with fighting in the past. Some were afraid to agree to participate in a health program associated with armed groups, as they expected negative reactions from competing groups:

*“When we hold a meeting to talk to the village chiefs about the program, we always tell them about the partners. Many are still afraid to be involved with the “rebel groups”, but actually the KNU is not classified as a rebel group by the central government anymore. The villagers have struggled in the war zone [sic], if they see a mix of different armed groups coming, they may be afraid that fighting will happen, they are still traumatised. We need to make villagers understand that they are safe if they accept the program.” (Area coordinator, m, 29).*

As Dickson-Gómez (2004) writes, *“For most people from the developed world, trauma represents something far removed from everyday life....However, those who were born in and reached adulthood in the context of war,..., have never experienced a world of safety*.. *.it leaves an everyday expectation of violence...”* ( Dickson-Gómez, 2004). In the case of the Thai-Myanmar border, violence had been present for more than half a century; and trauma had shaped the daily lives of those from Myanmar ( Tangseefa, 2006 and Tangseefa, 2016). With villagers having recent memories of violence, it was important that the CE teams always mentioned that they had permission from all local authorities to conduct the program in the village. This was to assure villagers that they would not breach with any authority if they participated in the program.

Moreover, because of the history of violence, many Karen/Kayin people in the program area distrusted the central government. Some local leaders would have rejected the program if it had been coming through the government, due to their own resentment or their inability to guarantee government staffers’ safety in the area. Government or government-associated INGO health programs had been set up very soon after increased ceasefire stability, while relationships between the government and the Karen/Kayin - leaders as well as villagers - were still not existing in many parts of the METF project areas. The only contact villagers in such areas had had with the government in the past was with the Tatmadaw (the Myanmar name of the Myanmar army) in the context of war. Government health staff for instance, who had been deployed to remote villages were reportedly often not familiar with local culture(s), language(s) and life in a rural area. Some of these health workers were described to often be absent and reluctant to approach local authorities and villagers and vice versa. Moreover, recent attempts of health and other INGOs to establish programs in cooperation with the government in the area had partly fed into distrust due to not being very sustainable or careless program conduct. One example named by different METF workers from different areas was a filariasis MDA program, which had reportedly been conducted without sufficient preparation of villagers and no follow up after MDA, leading to confusion and anxiety about side effects. Some people feared they had been poisoned and would die soon.

Aware of some local leaders’ concerns about government personel, METF workers usually mentioned that the program was conducted by Karen/Kayin health organizations. Moreover, except one, the METF members themselves were Karen/Kayin and hence of the same ethnicity as most villagers - some of the staff however were “inside outsiders” who had little experience of many parts of the project area. In order to become more similar to the villagers (and less similar to governing actors), the CE team members also adopted symbols of rural peoples’ cultures, like chewing betel nut, wearing thanaka powder (stem bark powder of *Hesperethusa crenulata*, which is applied as a cosmetic in Myanmar) or a village farmer’s hat. Approaching villagers as ordinary persons, as opposed to professionals or leaders, could sometimes make the latter feel more comfortable.

The lack of affiliation with governing actors could be exacerbated by the geographical isolation of some villages due to the mountainous terrain and lack of infrastructure. Leaders and health care providers had weaker or no ties to populations in such areas. Moreover, in some villages that were at the borders between two or more spheres of governing actors or leaders, the central governing actor through which the METF had received permission and support to approach villages, was not de facto in power. Sometimes, such villages had stronger ties to a bordering leader or armed group, for instance when the villagers felt more supported by the bordering leader or were disappointed by their area’s leader. In such a village, it could be that the METF came “through the wrong person”.

*Leveraging or building trust*

In such an environment of distrust, it was crucial that the METF either came through trusted leaders or built trust as a main task of CE. As part of its make-up as a task force, the METF leveraged trust relationships and authority by employing local trusted authorities, informal leaders or healthcare providers, who had already cared for villagers during the past. If a leader had authority, villagers were more likely to follow his/her request to come to a mass meeting where the METF was introduced or participate in program activities more generally. Health care providers often were positively associated by villagers. As one zone coordinator stated:

*“We explain that we are not doing this program alone, but with many partners: KDHW [Karen Department of Health and Welfare], MTC [Mae Tao Clinic], BPHWT [Backpack Health Worker Team]. Most people know Dr. Cynthia [founder of MTC], she is famous, some have been to MTC before.” (Zone coordinator, m, 49).*

A focus group of survey staff stated:

*“People cannot remember the name ‘METF’, but they remember the people. So when we talk about the program we call it ‘The program that [name of local medic] is doing’. Then they understand which program we are talking about.” (Focus group of five METF survey team members).*

If the person associated with the program was a trusted person who had done good for villagers in the past, villagers did not primarily participate due to their understanding of the purpose and benefits of the program, but also due to a culture of obedience and obligation to seniors and leaders whom they trusted ( Box 2). Such leaders, or in a broader sense, influential persons could also be found in a village. They were influential due to various reasons: They had a leadership position in the village, experience with the outside world, been educated in towns or so-called “refugee camps” on the Thai side, were religious authorities, elders or providers of healthcare. Such persons were key for the team to engage with, as villagers listened to them and seeked advice from them. Moreover, they sometimes seemed to feel threatened in their status by the METF team and could be detrimental to the program if they spread a rumour. One CE strategy was to find out about such persons in advance of approaching a village, visiting them to show respect or even giving them a role to represent the program. Doing so would prevent undermining their authority and at the same time allow the team to leverage their influence for CE.

Box 2. **Types of leaders and influential persons**

- Health workers from KEAO health departments and community based health organizations, community health workers
- Traditional healers, informal healthcare providers
- Village head persons
- Religious leaders like monks
- Teachers
- People who had experience with the outside world and/or education
- Seniors/Elders

However, in some villages, it was very difficult to approach villagers through leaders, for instance if they lacked authority or were not supportive of the program. some villagers would show no response to the program introduction, express no questions and concerns. Such conditions made it difficult to begin a conversation about the program through the formal approach of a mass meeting, usually supported by local leaders. As a CE worker told us:

*“Some people came to the mass meeting, actually the number of participants was ok. They listened, but there was no response, no good, no bad, no anything. But then only 45% came to participate in the MDA.” (CE worker, f, 41).*

In such villages, the “horizontal CE” activities were a way to get in touch with villagers and to build trust ( Box 3). We use the term *horizontal* to describe CE practices through which workers were approaching villagers informally on a personal or household level. Through such practices, CE staff tried to create *safe spaces of engagement*, in which villagers would be more comfortable to ask questions and express concerns. Where workers did not make an effort to spend time with people or spent too little time due to time pressure, participation would often be low. The importance of small group discussions and home visits for making people feel free to speak was emphasised by several staff. Such small group discussions were most successful, when they were emerging from an informal path:

Box 3. **Examples of “horizontal” community engagement**

- Spending time with villagers, listening to them
- Visiting villagers at home
- Having a meal with villagers
- Joining villagers in their work (around the house, on the farm)
- Attending and helping to prepare celebrations, weddings, funerals, and religious practices
- Showing films on a little TV
- Playing with children

See Kajeechiwa *et al.* (2017) for a detailed reflection on their CE activities

*“When we do small group discussions, we don’t say “please come here at this time”, we don’t make an appointment. We pick a house from the map that is in a cluster (that is why our self-drawn walking map is very important), go there and make a nice atmosphere, then let the people from that house call villagers from surrounding houses. We ask them from which house they are, how many people are living there. Only after that we start talking about MDA.” (CE worker, f, 41).*

In order to approach all people in the village, it was important that members of different social groups were represented among the CE team, such as women and men, young and seniors. Related to social hierarchies, senior members were in the appropriate position to approach elderly people or those in a respected position.

The CE workers themselves had to be comfortable among the villagers and enjoy the mutual activities. They had to be comfortable with the food, be able to live and sleep in a village for some days and be fluent in sociable activities in the cultural context of the area. For each village, the METF tried to have a local health worker who knew the area or was from the village in the team. By integrating local health workers and influential persons in its team, the METF built a continuum between the outside program staff and people from the population.

Moreover, METF staff emphasised that activities that symbolised *caring* and *empathy* were helpful to build relationships with villagers. Caring was expressed in the interest the workers showed in villagers’ daily lives, the OPD care, snacks being provided with MDA, being there 7 days from the start of MDA in order to explain and cure side effects and following up on them at their homes after MDA. Moreover, staff sometimes had to listen to people for a long time who wanted to complain or talk about grievances. These examples of caring were appreciated by villagers, as many felt that they had not been cared for by governing actors during the war. In Carruth’s study on two health programs in the Ethiopian postwar setting of the Somali region, for example, she similarly names caring as one core aspect for building trust relationships with people from the population the program approached ( Carruth, 2016). The need for care also concerned the need for malaria and health care.

***3.2.3 Perceived need for malaria care.*** In isolated areas where health and malaria care were deficient, people had personal experience with the disease either themselves or in their social surroundings. Hence, they felt that there was a need for malaria care and were interested in the elimination of the disease. Many staff reported that when they introduced the METF in meetings with authorities, they usually reminded attendees of the burden malaria was posing on them:

*“In old times we did not have medicine, now we have medicine that can treat malaria effectively, our children will only hear of malaria, they will not face it anymore.” (CE coordinator, m, 48).*

Staff also went a step further and talked about how malaria affected villagers’ productivity. One zone coordinator gave an example of what he told villagers:

*“If your child or wife is not well, you need to take care of them, you cannot go to work. It will affect your income and your child cannot go to school - it wastes your time.” (Zone coordinator, m, age unknown).*

As most villagers were highly dependent on their physical ability to sustain their livelihood through farming or work, pointing out such secondary effects of malaria on productivity was very effective. However, there were some areas where people did not see the program as a benefit for their health or productivity. In those contexts, the staff were facing challenges to achieve program participation. There were various reasons why villagers would not participate.

In places that were isolated from health care, but where people had never received support from governing groups, some had adopted an attitude of self-reliance and toughness. This meant that they preferred to handle disease by themselves and only seek help if they were really badly sick, making it hard to convince them to participate in MDA at a time when they were perfectly healthy. The staff tried to change their mind by emphasizing the benefit of malaria elimination for the community: if malaria were eliminated, their children and following generations would never have to face the disease again.

Moreover, elderly people sometimes felt no personal need for the program. They were not eager to improve their health anymore, as they knew that they would not live long. Similar to the case of independent people, the METF team tried to convince the elderly by directing their attention to the benefit for future generations. The staff associated their arguments with religious and social values, like purity before death or not causing harm to anyone.

Such arguments however did not convince comparatively affluent and business people or people from villages close to towns with privileged access to healthcare. Our study did not investigate this group of people deep enough, however the results from our previous study suggest that they did not feel in need of a free malaria program as they were able to access and purchase malaria care by themselves. Moreover, many business people were not from the villages in which they stayed and appeared to have a more individualistic mindset. Hence, they did not feel responsible for the health of the community or less affluent people ( Tangseefa *et al.*, unpublished report).

*Need for more than malaria care*

In most of the program areas however, people were not this privileged and there was a general lack of healthcare services. Malaria was only one of many health issues in these places and often became a minor concern after MDA. Hence, one shortcoming of the METF was that it only targeted malaria. The METF could not compensate for this lack of healthcare services. Moreover, the installation of a MP in a village without a health care provider could be problematic:

Case 3 - In one village the MP worker, who also had completed a Community Health Worker training, had started to treat minor illnesses as well, as people did not only come to him for fever, but for any kind of health condition. He had to buy medicines from a local shop to treat them and if they did not have any money, he had to pay for those by himself. As he had no extensive medical training, he sometimes phoned his brother who was working as a medic, to help him diagnose conditions he was not sure about. (MP worker, m, 27).

The example raises the question whether an isolated malaria program is problematic in a setting where healthcare is deficient. In many villages, MP workers had raised their interest to be trained as community health workers and be allowed to also treat other conditions beyond malaria. Particularly after MDA or prolonged MP existence, malaria cases dropped and MP workers started to feel irrelevant to the needs of the villagers. In order to respond to this demand, the METF had started to provide a few extra services (such as tuberculosis detection) through support of local clinics in order to maintain its relevance to people’s needs after malaria prevalence declined.

## 4 Conclusion

The challenges to generate participation and strategies reported by METF workers resonated with several aspects named by Sahan *et al.* (2017) and Kajeechiwa *et al.* (2016) and Kajeechiwa *et al.* (2017). Sahan *et al.* (2017) for instance also mentioned the importance of obtaining approval with various governing actors, the role of village leaders in approaching and assembling villagers, the benefit of bringing basic health care, the importance of local healing practices or socio-economic factors and access to healthcare, trust building through commensality and participation in local social activities. In their systematic review, Adhikari *et al.* (2016) mention two strategies of CE that were also employed by the METF. Those were working through existing social structures including health service providers, and human resource mobilization - meaning the involvement of community members and health staff in the program. The METF did this through employing health leaders and leveraging their health care structures, as well as the involvement of local influential persons in the village. Incentives however, did not play a role in the CE approach of the METF. Moreover, the eight dimensions of people’s lifeworlds Tangseefa *et al.* (unpublished report) conceptualised during the METF piloting phase appeared as important aspects in this study again. Most importantly, people’s experiences with the outside world were related to what Tangseefa *et al.* named “People’s knowledge about the world” and the degree of isolation of the “space they lived in”. Leadership played an important role for the METF to enter the program area and approach and gain trust from villagers. The perceived need for (malaria) care as well as affiliation with health care providers resonated with aspects of affiliation with health institutions mentioned by Tangseefa *et al.* Moreover, rumours were again emphasised as tremendous to participation.

Our study shifted the focus to the way that CE responded to those notions of people’s lifeworlds. CE strategies had to: be politically sensitive, build mutual understanding between staff and villagers and establish a sense of authority and trust. The notion of mutual understanding entailed a core part of CE, which was learning about program areas and villages in advance in order to be able to approach and adapt the further CE activities to villagers’ lifeworlds. The staffers’ success relied on their existing relationships, knowledge, experience, social positions, connections, as well as the attitudes they had in the political sphere of the program areas and towards people and the latter’s lifeworlds. The results also showed that participation was partly determined by the high malaria burden and lack of access to health care. As malaria incidence drops after MDA and MP setup, the health needs of villagers will be dominated by other diseases, so the METF will have to consider how to sustain support for the program after malaria decreases. The program is facing a paradox: It needs to run to curb the malaria incidence. Yet, having been successful, concerned parties will be less interested in the program - which could cause malaria to increase again.

The overall conclusion from our study was that successful CE is highly context specific: CE success was based on the adaptability of the approach towards the complex political and socio-cultural contexts of the different areas of the Karen/Kayin State. Hence, CE is not a fixed approach or strategy defined before entering the setting, but constantly evolving based on the encounters of workers with stakeholders and people of the population.

### 4.1 Data limitations

The data for our study were *heavily* based on the perspectives of the METF staff. Interpretations regarding people’s lifeworlds are therefore mainly derived from the interpretation of strategies reported by the METF staff. The field researcher did not speak any of the Karen/Kayin languages and was thus dependent on the translation and interpretation of a translator. As the translators were also METF program staff, this may have biased the interviewees (staff and villagers) in their way of talking about their work and experience with the program respectively. When it came to participant observation, the researchers’ language limitation was an obvious issue. Moreover, due to difficult geographical terrain and limited usability of roads during the rainy season, as well as the political and safety barriers to travel, the field researcher had a very limited access to program areas, which minimised direct observation and interview opportunities with villagers.

We have only looked at short-term aspects of CE success. However, it is likely that the extensive education efforts the METF made to prepare local health workers and students for conducting program activities will have long-term benefits for health care in the program areas. Moreover, the facilitation of collaboration among leaders of various local armed groups may have a positive effect on peace in the program area. Another point is, that CE was not gender neutral. While a gender sensitive perspective goes beyond this study, it was obvious that female staff sometimes played particular roles in CE. It would be interesting to look at this aspect in the future.

## Data availability

Due to concerns that interview participants could be identified by the interview content, and concerns that information given in interviews could be damaging to the program or the interview participants in the sensitive political context of the program area if disclosed, data are not made accessible.

## Notes

^1^The authors of this report are Decha Tangseefa, Krishna Monthathip, Naruemol Tuenpakdee, Ladda Kajeechiwa, May Myo Thwin, Suphak Nosten, Andrea König, Lorenz von Seidlein, Phaik Yeong Cheah and Francois Nosten.

## Supplementary material

**Supplementary File 1: Guiding interview questions for semi-structured interviews and FGDs with METF program participants.**

Click here to access the data.

**Figure S1: METF Organizational Structure.** This figure shows the METF organizational structure including all staff positions within the METF. The brown fields show members of the executive committee, while the blue fields show operational or field staff. Acronyms: SMRU = Shoklo Malaria Research Unit, CPI = Community Partners International, MAM = Medical Action Myanmar, KDHW = Karen Department of Health and Welfare, KNU/KNLA Peace Council, KBGF = Border Guard Force in Karen/Kayin State, KKO = Klo Htoo Baw Karen Organisation, MTC = Mae Tao Clinic, BP = Backpack Health Worker Team, BMA = Burma Medical Association, Asst = assistant, T&M = training and monitoring, Coord = coordinator, mgmt = management, MP = malaria post.

Source: The figure was provided by the METF with the permission to display it in this publication.

Click here to access the data.

**Figure S2: METF Area Organization.** Chart 2 shows the organizational structure of staff working within the METF project areas. Acronyms: Coord = coordinator, Asst = assistant, Fin = finances, MP = malaria post, Superv = supervisor.

Source: The figure was provided by the METF with the permission to display it in this publication.

Click here to access the data.
