# Supplementary material for: Community engagement for malaria elimination in contested areas of the Karen/Kayin State, Myanmar: A case study on the Malaria Elimination Task Force
Source: Wellcome Open Res. 2018 Mar 6;3:22. [Version 1] doi: 10.12688/wellcomeopenres.13965.1 (PMC13570122; doi:10.12688/wellcomeopenres.13965.1)
Supplement: Peer review history

# Reviewer report: Reviewer response for version 1

**DOI:** 10.21956/wellcomeopenres.15179.r31538

**Published:** 2018-04-13

**Authors:** Christopher Lyttleton

**Affiliations:** 1 Department of Anthropology, Macquarie University, Sydney, NSW, Australia

The article advances a useful analysis of the importance of community engagement (CE) in health programs and the ways to achieve this.  It argues that careful CE, in particular the development of trust, is necessary in order to maximise the benefits of public health interventions, in this case attempts to eliminate malaria. The article focuses on the strategies used to introduce mass drug administration (MDA) in villages in (ex-) conflict areas in Eastern Myanmar where issues of distrust and political sensitivity to outside intrusions are elevated due to decades of armed conflict. Of particular value, to my mind, is the emphasis on local world-views and the need for adequate mutual understandings as a precondition to introduction of new health practices.  It also advances the important point that effective deployment of CE in different settings needs to take into account variable cultural, historical and political factors.

 This is a salient reminder to those working in malaria, or health programs more generally, among marginal populations. Cultural sensitivity has long been recognised as a crucial dimension to the global roll-out of health initiatives, but it is remarkable how often it falls to the wayside on the assumption that scientific benefits of biomedicine will be self-obvious to all. A case in point is a recent article in *Malaria Journal* written by reputable malariologists assessing the use of Mass Drug Administration in a Lao province. The article describes the targeted villagers as belonging to the ‘ *Lao theung* ethnic minority’ as if this was a specific group’s name rather than common (sometimes perjorative) umbrella term for a large number of different ethnic groups in Laos.  One assumes it was an editorial slip, but the fact that the miswording wasn’t picked up in any review also confirms the familiar observation that the primacy of the ‘science’ of anti-malaria work often excludes appropriate cultural awareness.

 Such bias is not evident in the paper under review. It argues that appropriate communication with local communities is crucial to the project of eliminating malaria and demonstrates that such engagement relies, at least in part, on adequate cultural knowledge of the target populations. As a research report from the field, the article advances our knowledge of the ways effective public health strategies need culturally sensitive planning, consultation and execution. This is particularly relevant given the growing acknowledgement that total malaria elimination in the Greater Mekong Subregion (GMS) is the only way to deal with multi-drug resistance. Likewise local, often remote, communities (which in the GMS are typically ethnic minorities, migrants or other marginalised groups) are increasingly the focus of tailored interventions and, as such, discussions of how to work together to combat resistance are vital steps. Too often malaria-affected populations are depicted as unreliable recipients rather than active participants and this article is a welcome corrective. That said, there are shortcomings in the article’s current formulation that limit its value in advancing these insights within a larger academic framework. These primarily derive from the way the conceptual argument is developed and justified.

 First, the article cites community engagement (CE) as crucial to malaria elimination. This is indisputable. It then suggests that literature on “the role and nature of CE in such contexts is scarce” (pg 3). This might be technically true given the recalibration from control to elimination is relatively recent in the GMS, but it is a very partial view. Notwithstanding the fact that eradication was forefront in anti-malaria campaigns in the 1950s and 60s, this perspective ignores the widespread work in malaria control over more recent decades wherein community engagement was, and continues to be, a central platform in ways that overlap with elimination approaches. There are many publications dating back to the 1990s (and before) advocating community participation in anti-malaria work, including near the Myanmar border. The current article mentions in passing the establishment of malaria posts (and volunteers) as one of its own program components. Yet it makes no reference to the fact that this initiative is built around community engagement and has been employed in numerous settings; nor does it mention the wealth of literature assessing strengths and weakness of community approaches and examination of associated socio-cultural determinants of malaria risk. Inclusion of material detailing broader relevance of, and experiences with, community participation in anti-malaria work would ground the argument more persuasively.

 Leaving out examination of various modalities of CE in anti-malaria programming, the article focuses on MDA as the necessary step towards elimination. The article implies that MDA is an obvious and urgent initiative to halt the spread of drug-resistant malaria in the GMS and elsewhere.  This position needs to be validated rather than assumed. WHO recently consigned an evidence review group on the role of MDA to ascertain its viability. The review concluded that “In the absence of sufficient evidence, WHO does not recommend the use of MDA in situations other than for areas approaching elimination, epidemics, and complex emergencies, as specified above” (MPAC 2016 ^1^). The article should clarify how, and to what extent, the target villages fit WHOs specific criteria. It may be that the described project will deliver convincing data to assist delineation of where MDA is most applicable. But, at the same time, there are examples where MDA has created adverse consequences both in terms of its well-documented association with chloroquine resistance and present-day examples of community resistance prompting uneven uptake. In other words, MDA is the subject of important debates. The authors should alert the reader of these polemics and their implications rather than by-passing them.

 Third, even assuming there is irrefutable data that demonstrates the role of MDA in protecting global health security, care needs to be taken that the ways cultural sensitivity is advocated as a means to advance uptake does not replicate other examples where ethnographic exploration became a tool to effectively remove community resistance. Again, there is long history of medical anthropology being recognised as a useful component of global health programs. But there is also criticism that community engagement can be co-opted to improve the effectiveness of the intervention (ie the spread of biomedicine), rather than for genuine participation or exchange of views. As above, these are relevant debates, which could be usefully discussed given that MDA is arguably as much about pre-empting a global threat as it is about solving a local problem (the article mentions some communities consider the malaria program to be of little benefit). 

 I suggest the first two of the above concerns be addressed in a revised version of this article, in particular demonstrating a more robust engagement with existing literature. By doing so the article could be made more persuasive and of optimal use to the reader. I raise the third concern for the authors’ reflection, but I accept the point that it would change the tenor of the article as it currently stands. Therefore, depending on the authors’ inclination, I would regard its inclusion in a revised version as desirable but not essential.

Is the work clearly and accurately presented and does it cite the current literature?

Partly

If applicable, is the statistical analysis and its interpretation appropriate?

Not applicable

Are all the source data underlying the results available to ensure full reproducibility?

Partly

Is the study design appropriate and is the work technically sound?

Yes

Are the conclusions drawn adequately supported by the results?

Yes

Are sufficient details of methods and analysis provided to allow replication by others?

Yes

Reviewer Expertise:

Medical anthropology, qualitative research

I confirm that I have read this submission and believe that I have an appropriate level of expertise to confirm that it is of an acceptable scientific standard, however I have significant reservations, as outlined above.

## References

[1] : Malaria Policy Advisory Committee to the WHO: conclusions and recommendations of eighth biannual meeting (September 2015). *Malaria journal* .2016;15(117) :10.1186/s12936-016-1169-xPMC476663726911803

# Response

**Published:** 2026-06-12

**Authors:** Andrea König

**Affiliations:** Heidelberg Institute of Global Health, Heidelberg University, Heidelberg, Germany

*-Additional resources have been added to the manuscript section 1.2 Community engagement in the Malaria Elimination Context* *-* *The question of whether MDA was suitable in the study setting or not was not part of the present study. The scope was to describe the role of CE in this MDA setting. A statement to clarify this has been added to the manuscript.*

# Reviewer report: Reviewer response for version 1

**DOI:** 10.21956/wellcomeopenres.15179.r31536

**Published:** 2018-03-27

**Authors:** Javier Lezaun

**Affiliations:** 1 Institute for Science, Innovation and Society (InSIS), School of Anthropology and Museum Ethnography, University of Oxford, Oxford, UK

The paper presents a unique experience of community engagement (CE) in support of malaria elimination in Karin/Kayin State, along the Myanmar-Thai border. Conducted under challenging geographic and political circumstances, the Malaria Elimination Task Force (METF) hinged on the mass administration of drugs to eliminate *P. falciparum* malaria and combat the rise of artemisinin-resistance in the area. CE was an integral part of this effort. It was used to gain access to hard-to-reach and often isolated communities, and to maximize participation in the mass drug administration (MDA) component of the campaign. The paper reports on the experience gathered by members of the METF CE team in the course of their activities.

 There are several noteworthy aspects of the experience that come across in the paper and will be of relevance to malaria elimination efforts elsewhere. One is the adaptability of CE to very fluid political conditions, characterized here by multiple, partly conflicting and partly overlapping systems of authority, most notably those of the central Myanmar government and the different Karen ethnic armed organisations operating in the area. The authors make the obvious but important point that there is no ‘one-size-fits-all’ approach to community involvement in MDA-driven malaria elimination campaigns. As their material makes clear, moreover, the definition of what the relevant community is, and the unraveling of its multiple layers of authority and obligation, cannot precede the design of the intervention, but come into relief as that intervention unfolds. In this respect, the paper offers a useful example of how to build a reflexive CE machinery amidst an ongoing malaria elimination campaign. In this case, it is achieved by focusing on a set of locally specific “lifeworlds,” which are investigated as the elimination effort proceeds.

 The paper also offers glimpses of the solutions adopted for some of the most intractable challenges encountered by the METF. One is how to justify participation in the MDA to individuals who find no personal benefit in the drugs offered by the program. This includes the very elderly, but also the minority of individuals who have access to quality clinical care elsewhere. Another one is the tensions that are bound to emerge in a program that is narrowly focused on malaria elimination but is unable to address the manifold issues faced by communities with very limited healthcare provision. As the authors indicate, the example of METF raises the possibility of transitioning the infrastructure established for malaria elimination, specifically the staff directly employed by the programme, into other forms of healthcare provision (e.g. tuberculosis detection).

 Finally, the authors are to be commended for being very explicit about the limitations of their data. As they note, their account is reliant almost completely on the views expressed by METF staff. This is, in other words, a second-order observation of community engagement, in which the experiences and opinions of community members themselves are essentially missing. Moreover, most of the research was conducted in English with the help of translators, who were themselves members of METF. These limitations do not detract from the value of the paper, but qualify any conclusions regarding the level of voluntary community support for the program.

 Because of the broader relevance of this particular case to malaria elimination campaigns elsewhere, it would be important to address some of the shortcomings of the paper in its current version.

 First of all, it would be useful to introduce the political situation of Kayin State and the history of conflict in this section of the Myanmar-Thai border region in greater detail. Otherwise the reader unfamiliar with this context (e.g. this reviewer) will quickly get lost in the complex web of actors and acronyms. It is particularly difficult to grasp the key parameters of the situation as far as the competing sources of political authority is concerned.

 Second, the current paper provides no information about the criteria of inclusion/exclusion in the MDA. Were children included? If so, from what age? Do the authors have any insight into whether the decision to participate (or to not participate) was taken individually or collectively (e.g. at the level of the household, the village)?

 The paper very usefully discusses the rumours that in some cases impeded the effective roll-out of the program. Yet the only rumours and misapprehensions discussed in detail relate to the fear that taking blood (for diagnosis) might lead to an irreversible reductions in the amount of blood in the body. Historians and anthropologists who have analysed comparable biomedical interventions in other contexts often report a much wider set of anxieties around the extraction of blood for diagnostic purposes, including fears of blood theft and vampirism (White 2000 ^1^; Tappan 2017 ^2^). Similarly it would be useful to have a more extensive compendium of the anxieties generated by drug administration and how they were addressed.

 Third, how did CE activities for drug administration relate to other informational or educational elements of the campaign, specifically those relating to measures of individual protection (i.e. use of bed-nets, prevention of mosquito exposure when outdoors)?

 Fourth and finally, the authors mention, towards the end and only in passing, the relevance of gender to effective CE. This seems to be a (somewhat confusing) reference to the particular roles played by female staff in the METF. What is meant by this exactly? This is an issue worthy of further elaboration, and one that is directly relevant to the central argument of the paper.

Is the work clearly and accurately presented and does it cite the current literature?

Partly

If applicable, is the statistical analysis and its interpretation appropriate?

Not applicable

Are all the source data underlying the results available to ensure full reproducibility?

Yes

Is the study design appropriate and is the work technically sound?

Yes

Are the conclusions drawn adequately supported by the results?

Partly

Are sufficient details of methods and analysis provided to allow replication by others?

Yes

Reviewer Expertise:

NA

I confirm that I have read this submission and believe that I have an appropriate level of expertise to confirm that it is of an acceptable scientific standard, however I have significant reservations, as outlined above.

## References

[1] : Speaking with vampires: Rumor and history in colonial Africa. *University of California Press* .2000;37:

[2] : The riddle of malnutrition: the long arc of biomedical and public health interventions in Uganda. *Ohio University Press* .2017;

# Response

**Published:** 2026-06-12

**Authors:** Andrea König

**Affiliations:** Heidelberg Institute of Global Health, Heidelberg University, Heidelberg, Germany

Response to Reviewer Report 1 (Javier Lezaun)

 The paper presents a unique experience of community engagement (CE) in support of malaria elimination in Karin/Kayin State, along the Myanmar-Thai border. Conducted under challenging geographic and political circumstances, the Malaria Elimination Task Force (METF) hinged on the mass administration of drugs to eliminate *P. falciparum* malaria and combat the rise of artemisinin-resistance in the area. CE was an integral part of this effort. It was used to gain access to hard-to-reach and often isolated communities, and to maximize participation in the mass drug administration (MDA) component of the campaign. The paper reports on the experience gathered by members of the METF CE team in the course of their activities.

 There are several noteworthy aspects of the experience that come across in the paper and will be of relevance to malaria elimination efforts elsewhere. One is the adaptability of CE to very fluid political conditions, characterized here by multiple, partly conflicting and partly overlapping systems of authority, most notably those of the central Myanmar government and the different Karen ethnic armed organisations operating in the area. The authors make the obvious but important point that there is no ‘one-size-fits-all’ approach to community involvement in MDA-driven malaria elimination campaigns. As their material makes clear, moreover, the definition of what the relevant community is, and the unraveling of its multiple layers of authority and obligation, cannot precede the design of the intervention, but come into relief as that intervention unfolds. In this respect, the paper offers a useful example of how to build a reflexive CE machinery amidst an ongoing malaria elimination campaign. In this case, it is achieved by focusing on a set of locally specific “lifeworlds,” which are investigated as the elimination effort proceeds.

 The paper also offers glimpses of the solutions adopted for some of the most intractable challenges encountered by the METF. One is how to justify participation in the MDA to individuals who find no personal benefit in the drugs offered by the program. This includes the very elderly, but also the minority of individuals who have access to quality clinical care elsewhere. Another one is the tensions that are bound to emerge in a program that is narrowly focused on malaria elimination but is unable to address the manifold issues faced by communities with very limited healthcare provision. As the authors indicate, the example of METF raises the possibility of transitioning the infrastructure established for malaria elimination, specifically the staff directly employed by the programme, into other forms of healthcare provision (e.g. tuberculosis detection).

 Finally, the authors are to be commended for being very explicit about the limitations of their data. As they note, their account is reliant almost completely on the views expressed by METF staff. This is, in other words, a second-order observation of community engagement, in which the experiences and opinions of community members themselves are essentially missing. Moreover, most of the research was conducted in English with the help of translators, who were themselves members of METF. These limitations do not detract from the value of the paper, but qualify any conclusions regarding the level of voluntary community support for the program.

 Because of the broader relevance of this particular case to malaria elimination campaigns elsewhere, it would be important to address some of the shortcomings of the paper in its current version.

 First of all, it would be useful to introduce the political situation of Kayin State and the history of conflict in this section of the Myanmar-Thai border region in greater detail. Otherwise the reader unfamiliar with this context (e.g. this reviewer) will quickly get lost in the complex web of actors and acronyms. It is particularly difficult to grasp the key parameters of the situation as far as the competing sources of political authority is concerned.

**Response:** *Additional background information on the political situation of Kayin State and the competing sources of political authority has been added to the manuscript.*

 Second, the current paper provides no information about the criteria of inclusion/exclusion in the MDA. Were children included? If so, from what age? Do the authors have any insight into whether the decision to participate (or to not participate) was taken individually or collectively (e.g. at the level of the household, the village)?

**​​​​​​​Response:** *The missing information has been added to the paper in the introduction part 1.1.*

 The paper very usefully discusses the rumours that in some cases impeded the effective roll-out of the program. Yet the only rumours and misapprehensions discussed in detail relate to the fear that taking blood (for diagnosis) might lead to an irreversible reductions in the amount of blood in the body. Historians and anthropologists who have analysed comparable biomedical interventions in other contexts often report a much wider set of anxieties around the extraction of blood for diagnostic purposes, including fears of blood theft and vampirism (White 2000 ^1^ ; Tappan 2017 ^2^ ). Similarly it would be useful to have a more extensive compendium of the anxieties generated by drug administration and how they were addressed. **​​​​​​​Response:** *Interview participants were asked about anxieties and what they have responded is reported in the manuscript. Fear of vampirism was not mentioned. However, it is possible that these kinds of fears exist and have not been reported.*

 Third, how did CE activities for drug administration relate to other informational or educational elements of the campaign, specifically those relating to measures of individual protection (i.e. use of bed-nets, prevention of mosquito exposure when outdoors)?

**​​​​​​​Response:** *Yes, these were part of the health and medical concept educational activities of CE. A sentence for clarification has been added to the manuscript.*

 Fourth and finally, the authors mention, towards the end and only in passing, the relevance of gender to effective CE. This seems to be a (somewhat confusing) reference to the particular roles played by female staff in the METF. What is meant by this exactly? This is an issue worthy of further elaboration, and one that is directly relevant to the central argument of the paper.

**Response:** *This sentence at the end was added to state that during the interviews it became apparent that gender mattered for CE. However, the researchers did not look at or collect the data with a gender sensitive lens, nor were there sufficient examples of such roles to make any generalizations. We suspect that it does play an important role which could be researched by further study.*
